# A patient’s perspective on care decisions: a qualitative interview study

**DOI:** 10.1186/s12913-023-10342-9

**Published:** 2023-12-01

**Authors:** S. Briedé, O. N. Brandwijk, T. C. van Charldorp, H. A. H. Kaasjager

**Affiliations:** 1 Department of Internal Medicine and Dermatology, University Medical Centre Utrecht, Postbus 85500, Utrecht, GA 3508 the Netherlands; 2 Department of Languages, Literature and Communication, Faculty of Humanities, Utrecht University, Utrecht, the Netherlands

**Keywords:** Care decisons, Treatment decisions, Patient-physican communication

## Abstract

**Background and Objectives:**

Discussing treatment wishes and limitations during medical consultations aims to enable patients to define goals and preferences for future care. Patients and physicians, however, face multiple barriers, resulting in postponing or avoiding the conversation. The aim of this study was to explore an internal medicine outpatient clinic population’s perception on (discussing) treatment wishes and limitations.

**Methods:**

Semi-structured interviews were conducted in two rounds with 44 internal medicine outpatient clinic patients at the University Medical Centre Utrecht, a tertiary care teaching medical centre in the Netherlands. Interviews were transcribed verbatim and thematically analysed with a phenomenological approach and inductive, data-driven coding.

**Results:**

Four themes were identified, two (1–2) represent a deep conviction, two (3–4) are practically oriented: (1) patients associate treatment wishes and limitations with the end-of-life, making it sensitive and currently irrelevant, (2) patients assume this process leads to fixed choices, whilst their wishes might be situation dependent, (3) treatment wishes and limitations are about balancing whether a treatment ‘is worth it’, in which several subthemes carry weight, (4) the physician is assigned a key role.

**Conclusion and practice implications:**

The themes provide starting points for future interventions. It should be emphasized that care decisions are a continuous, dynamic process, relevant at any time in any circumstance and the physician should be aware of his/her key role.

**Supplementary Information:**

The online version contains supplementary material available at 10.1186/s12913-023-10342-9.

## Key points

Four themes were identified in the perception of an internal medicine outpatient clinic population’s on (discussing) treatment wishes and limitations. The themes provide starting points for future interventions. It should be emphasized that care decisions are a continuous, dynamic process, relevant at any time in any circumstance and the physician should be aware of his/her key role.

**Supplementary Information:**

The online version contains supplementary material available at 10.1186/s12913-023-10342-9.

## Introduction

“Discuss care decisions when discussing treatment with patients.” This is one of the ten Wise Choices compiled by the Dutch Association of Internal Medicine [1], to improve the quality and efficiency of healthcare in the spirit of the global Choosing Wisely campaign [2, 3]. Care decision discussions comprise a broad spectrum of topics, all with the purpose to align treatment with the preferences of the patient. This includes code status documentation (i.e. whether limitations to specific life-sustaining treatments are in place) and all forms of advance care planning.

Although the Wise Choice above implies care decisions discussions should be a regular part of the medical consultation [1], both physicians and patients face multiple barriers in doing so [4–9]. Patients face difficulties such as lack of knowledge, passivity and refusing to think about the end-of-life [5]. Maybe even more important is the patient’s unawareness of the relevance of care decisions and the expectation that physicians will initiate the discussion when needed [8]. Avoidance by both parties results in care decision discussions not taking place [9], and therefore an opportunity to improve the efficiency and quality of healthcare is missed.

Patients and physicians often perceive the care decisions discussion in the outpatient clinic as being too soon [4, 5, 10]. However, the quality standards of the Dutch association for Internal Medicine demand a code status is documented in every admitted patient [11]. Therefore, when discussions about care decisions are postponed, it could be the case that code status suddenly has to be discussed in acute settings (e.g. at the emergency department), when there is less time for discussion and thoughtful consideration of the patients preferences before making a decision. Besides, in acute settings pre-existing physician-patient relationships are rare [5]. Therefore, the outpatient clinic setting would be a better option [12, 13].

There are some subtle differences in the Netherlands between hospitals and settings in how one refers to care decisions. Terms that are for instance used are: treatment restrictions, code status discussions, advance care planning (often associated with end-of-life) [4] and treatment instructions. In the communication with patients we used the term ‘treatment wishes and limitations’, as this makes clear that the discussion about care decisions is **not** just about a code status or treatment restriction, but about patient preferences and aligning treatment with these preferences, in which refraining from a treatment is also a possibility.

To stimulate and improve care decision conversations at the internal medicine outpatient clinic, we previously conducted a study in which internal medicine physicians were trained on the topic of care decisions and patients were computer-randomized to receive a patient education on this topic [14]. Care decisions were not specifically related to current treatment of these patient, because the intention was to improve timely care decision discussions in all patients. This patient education was constructed based on expert opinions and in collaboration with a patient panel. The patient education is an online web page that emphasizes the relevance of discussing care decisions and provides background information, for instance about who can initiate the discussion (patient or physician), who can decide on treatment limitations, how this is documented/ for whom this information is available. Examples included treatment wishes for patients for ventilation at the intensive care unit or blood transfusion. Additional information, for instance about certain treatments and its consequences, is accessible through hyperlinks. Remarkably, patients assessed the patient education as informative and with good overall marks (median 7 out of 10), while at the same time not valuing it as helpful in forming an opinion about care decisions or discussing them [14]. To clarify this contradiction and gain insight in what would be more helpful for patients to aid them in care decision conversations, we conducted the current study.

Most research on treatment wishes and limitations and advance care planning is conducted in end-of-life settings [8]. On the other hand Harris et al. conducted a qualitative interview study on goals of care discussions in acute hospital admissions in Australia [15]. Knowledge about the perceptions of a relatively healthy outpatient clinic population towards this subject is lacking.

We used semi-structured interviews to (1) further evaluate our patient education, (2) gain in-depth insight in patient’s perspective on the topic of care decisions, and (3) gain insight in necessities (from the patients’ view) for discussing care decisions. In this article we focus on the results of the last two, as these insights are most relevant for a wide audience. Our results should enable us to improve patient education and discussion of care decisions.

## Methodology

### Study design

This study is part of a larger project, aimed at stimulating and improving care decision conversations at the internal medicine outpatient clinic of the University Medical Centre Utrecht, a tertiary care teaching medical centre in the Netherlands. We conducted a descriptive qualitative study with a phenomenological approach to explore patient’s perspective on the topic of care decisions and the patient education.

This study was approved by the Medical Ethical Committee of the University Medical Centre Utrecht (MEC 18–465). The study procedures comply with the Declaration of Helsinki. The study was reported using the consolidated criteria for reporting qualitative studies (COREQ) (see supplementary appendix 1) [16].

### Participants

We used convenience sampling to select participants that had received the patient education in a clinical setting (i.e. the intervention group of the previous study). In this previous study, patients over the age of 18 with a scheduled outpatient visit with a participating physician, were eligible for inclusion. Physicians belonged to the department of internal medicine, nephrology, gastroenterology, endocrinology, immunology or vascular medicine. Exclusion criteria were: inability to read Dutch, inability to give informed consent, or recently (< 2 years) documented treatment limitation discussion. All patients that gave permission to be approached for further research-questions, were contacted for this particular study. They were informed about this subsequent interview-study by phone, and asked for verbal informed consent. When verbal informed consent was given, interviews were planned and executed by phone.

**Fig. 1:**
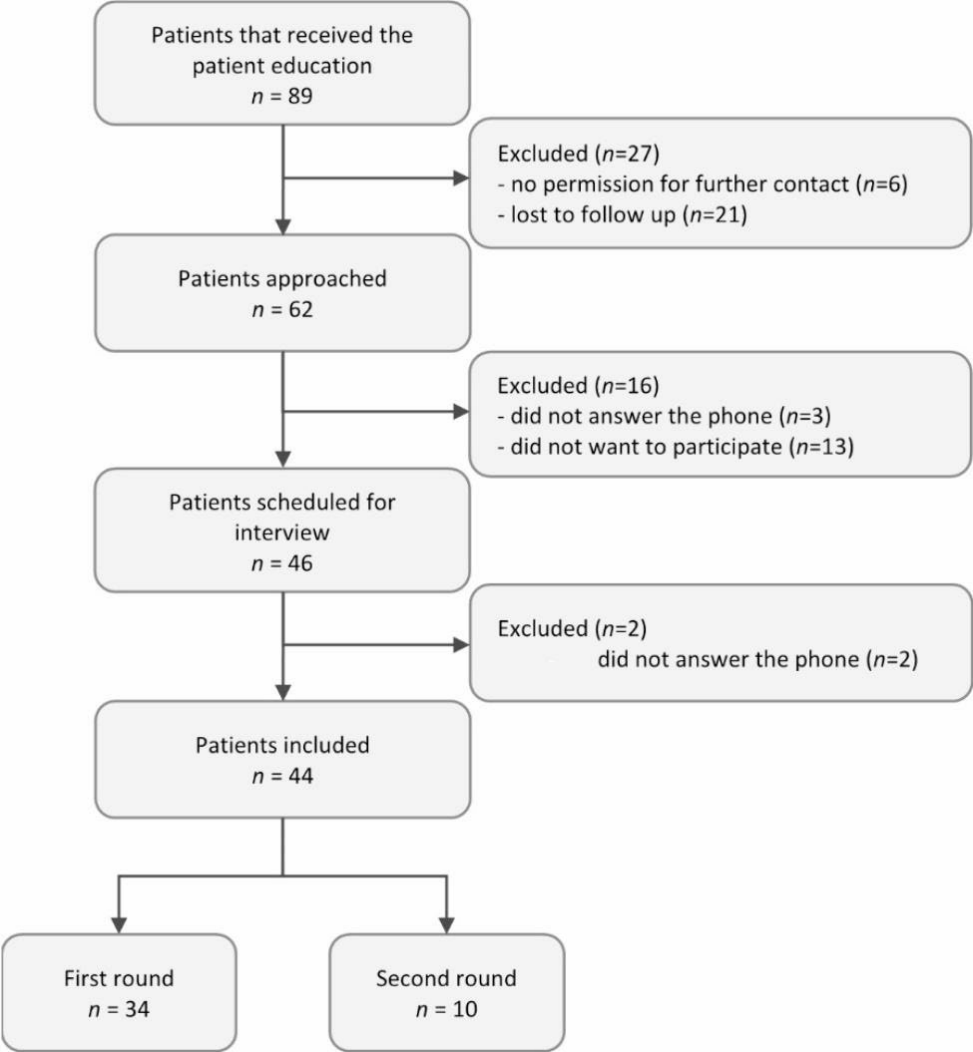
Diagram of the participant flow

Figure 1 shows the inclusion of patients. In two rounds, a total of 44 patients were interviewed, 34 patients in the first round and 10 in the second round. Interviews were conducted in two rounds for both practical and methodological reasons: the former study was not ended yet during the first round of interviews, so new eligible patients were available after the first round, and this gave us the opportunity to adjust our interview guide in-between the rounds based on our first analysis. One interview was only partly recorded due to a technical issue. Of the 44 patients, 25 were male (56,8%) and 19 female (43,2%). The median age was 57,5 years (interquartile range 53–67,5) and they had a median Charlson Comorbidity Index of 2,5 (interquartile range 1–4) [17].

### Data collection

The semi-structured interviews were conducted for the purpose of this study in two rounds, January 2020 and June/ July 2020, by two medical students (AB and NB respectively) after training. The interviewers did not have a former relationship with the participants and were not involved in the recruitment of patients in the previous study or the distribution of the patient education, to ensure patients could be as honest as possible in their evaluation. The interview guide contained straightforward questions about the content of patient education and open and flexible topics regarding feelings and expectations, allowing new or unexpected responses to be introduced (see supplementary appendix 2). In between the two rounds, the first set of interviews was analysed and the interview guide was adjusted according to these results. Main adjustments were removal of design-related questions (as we had sufficient input on that topic) and additional questions regarding patient perspective on the topic of care decisions, to explore this further. Interviews were conducted by phone to minimize burden for the participants and audio-recorded.

### Data analysis

All interviews were transcribed verbatim, anonymised and coded using NVivo 12 software. Collected data were analysed using reflexive thematic analysis with an inductive approach, meaning that the process of coding was data-driven [18–20]. Two authors (SB & NB) independently familiarised themselves with the data by reading and re-reading all transcripts. We used an iterative and flexible coding process. SB and NB identified, discussed, refined and revised codes regularly and when necessary a third author (TvC) was consulted until full agreement was reached. First theme development took place in multiple sessions with SB, NB and TvC with use of visual mapping to aid pattern formation and identification. In additional sessions with all four authors, themes were reviewed and refined. Throughout the process, we operated within a qualitative paradigm, corresponding to the “Big Q thematic analysis” described by Terry et al. [19] and kept the research questions in mind. Opposed to “small q thematic analysis”, often used in positivist research, “big Q thematic analysis” is characterised by theoretical independence and flexibility, and organic processes of coding and theme development. “*The researcher is more like a sculptor, chipping away at a block of marble. The sculpture is the product of an interaction between the sculptor, their skills and the raw materials. Analysis becomes a creative rather than technical process, a result of the researcher’s engagement with the dataset and the application of their analytic skills and experiences, and personal and conceptual standpoints*” [19]. In the later stages of theme development, we moved to an interpretative orientation and used thematic maps to gain a deep understanding of the dataset to identify and understand potential themes in relation to each other and the overall dataset. In the final stages of the analysis, after data sessions with all authors, we constructed our final model, that captures the relations and connections within our dataset and provides an answer to our research questions.

## Results

In this section, we show our thematic map, followed by a narrative clarification of this map, a table with examples from the interviews and a description of the themes and how they relate to one another.

**Fig. 2:**
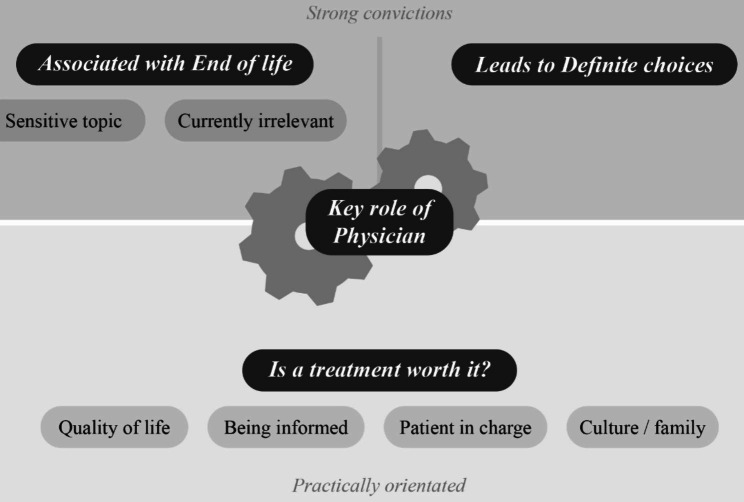
Thematic map of patient perspective on care decisions

Figure 2 shows our thematic map. It is important to point out that two themes at the top layer in Fig. 2 represent strong convictions of what patients associate with care decisions, whilst the other two themes are more practically oriented. The two strong convictions are: (1) that discussing care decisions is about the end-of-life phase and (2) discussing care decisions leads to fixed choices. The end-of-life association results in the perception that care decisions are a sensitive topic, and irrelevant for the patient at *this moment in time*. The perceived ‘definiteness’ makes some patients hesitant towards discussing the subject. One of the more practically orientated themes is: (3) (when relevant) care decisions are about balancing whether a treatment is ‘worth it’, in which several subthemes carry weight: quality of life, culture/ family, being informed and patient in charge. The final (also more practically orientated) theme is (4) the physician. Patients assign the physician a key role in the care decision process. To show this important position we visualized the physician as the wheel that moves the process along.

Table 1 shows illustrative quotes for each (sub)theme taken from the interviews. We dig deeper into these themes and the connected subthemes in the following paragraphs.

**Table 1:** Quotations illustrative for the theme’s

End of life	“Oofh (laughter) yes then I think about the last phase (…) suppose you are terminally ill or there is no more treatment possible, then you start thinking about this stuff (…) Yeah (laughter) I- you don’t start thinking about this stuff when you’re healthy and when there’s nothing wrong” (37)
Sensitive topic	“I walk away from that kind of sensitive subjects” (12) “otherwise I think people feel like oh why do I receive this, why does he say that, ouch” (40) “Because it’s about a precarious thing, you can die or not, do you want to be resuscitated or not.” (39)
Currently irrelevant	“it was not really relevant, it did not come up, because I visited for a silly cholesterol-story and there was nothing life-threatening about that (…) I can imagine if I ever get a diagnosis like madam you have cancer and it can take a few more years but, etcetera. That then I would think about things like that” (36)
Definite choices	“it depends on many factors and how you are at that moment and what happens, to record this now already I think noh” (40) ^a^ “I would not want to have answers to this recorded in my medical file because you can absolutely not oversee how you would react in certain circumstances or would like to react” (5)
Is a treatment worth it?	“you state your own boundaries for whether you want to be treated and what is acceptable concerning side effects and what not” (43)
	“it depends on many factors and how you are at that moment and what happens, to record this now already I think noh” (40) ^a^
Quality of life	“but if the quality of life is so low then I think you should have the right to say well until here, no further” (42)
Being informed	“Of course I am unaware of what all possibilities are and what its limitations are”(28) “But I think it is nice to be able to put the possibilities in context” (1) “I think that it starts with such a patient education, through which eh based on the information you receive eh yes actually get the knowledge to first check for yourself, gosh what is my point of view, how do I think about that” (41)
Patient in charge	-) “I think anyway regardless of the disease also diabetes or other diseases it is important to think about what you want for yourself and what you do not want and make that a topic of discussion” (26)
Culture/ family and loved ones	“But if it is that moment, and maybe you are too late, then my husband should decide (…) he knows very well what I want and what I don’t want” (7) “it is something you together, if you make a decision your family has consequences of that as well (…) see my father is a kidney-patient and he dialyses three times a week (…) so, he has a do not attempt resuscitation. He did discussed that with us and we respect that”(18) Because unexpectedly you can get into an accident resulting in being unconscious and then somebody else should know how you want it”(33)
Physician	
Open and sensitive communication	“In -if possible but yeah those doctors are not all equally empathic and you name it- but in a friendly, calm and clear way” (23)
Physician initiates	“what comes to my mind is that it is well how should I say it a necessary topic could be what comes up in the conversation with a physician the moment there is a reason for it eh yeah given the disease development of the patient” (41) “because then the physician makes it discussable, because probably a lot of patients are not thinking about this yet”(15)
Discuss	“I think that is important I think you have to know what the options are but also that the doctor knows how you feel about it”(1)
knowledge deficit	“You really need the doctor for that (…) I have no idea about that and I don’t know what the costs of that are, the costs in pain and in discomfort it gives the patient for example” (28)

^a^Some quotes relate to more than one (sub)theme, as for example the quote marked with an Asterix from interview 40 relates to both *definite choices* and *is a treatment worth it*?

### End of life

Most patients shared a deep conviction that care decisions are about the end-of-life. They associated it with ‘a certain age’, ‘a bit older’, ‘terminal cancer’, ‘terminal illness’, ‘your last phase’, ‘the end-of-life’, ‘a severe illness’, ‘*very* sick’, ‘dying’, ‘life-threatening’, a bad diagnosis or prognosis’, ‘people who are worse’, and so on. As can be seen in these examples, some patients explicitly connected care decisions to the ‘last phase’ of life, or a certain illness. Others described it more vaguely as ‘something for later’, without being able to exactly indicate when ‘later’ will be. This basic attitude towards care decisions, i.e. it belonging to the end-of-life, results in two subthemes: sensitive topic and currently irrelevant.

#### Sensitive topic

Patients described the topic of care decisions as a sensitive topic. Because they associated the topic with the end-of-life and dying, they characterized it as ‘difficult’, ‘not a fun topic’, ‘precarious’, ‘a tricky subject’, ‘sensitive’, ‘heavy’, ‘loaded’, ‘confronting’ or ‘threatening’, and articulated feeling hesitant to talk about it. Patients expressed this is especially the case when it is unclear to them why the subject is discussed, or why they received the patient education. This makes sense in the light of the end-of-life association: when the topic is brought up, the patient thinks this implies they are at the end-of-life. In this way it might give the impression of being sicker than expected or getting an unfavourable test result, which can make them anxious. A few patients assumed discussing care decisions was (mainly) about whether or not to resuscitate, which could be contributing to the sensitivity of the topic.

#### Currently irrelevant

Patients perceived care decisions as a ‘far-flung’ event. The majority of patients identified care decisions as not relevant for themselves, at least not at *this* moment in time, because they did not consider themselves in that phase of life. They described it as ‘not really necessary yet’, ‘just not relevant’, ‘does not apply’, ‘ it is not on the agenda at the moment’, ‘too early’, ‘don’t really care too much about it yet’. The exact moment they believed it does become personally relevant varied. This is best summarised as: ‘when it gets a little closer’, and can be explained by the fact they related it to the end-of-life. This perceived current irrelevance is reflected in the evaluation of the patient education: patients assessed it as unhelpful in forming an opinion or discussing care decisions, because they felt they did not *need* to form an opinion or discuss care decisions yet.

### Definite choices

The second theme that represents a deep conviction is definite choices. Thinking about care decisions, discussing and talking about it and making choices seems to be irrevocably linked to the definite documentation of these choices in the electronic health record. Some patients saw this as an advantage: the physician is aware of the patient’s point of view in case of an unexpected situation in which the patient cannot express his opinion. On the other hand, however, several patients were hesitant towards discussing care decisions with the physician, because they were afraid this resulted in a fixed, documented decision. How you make up the balance might be situation dependent and varies over time. They expressed that ‘you push your limits’, and felt unable to decide now what they would like in the future: ‘how your condition is at that time and what happens’. This altogether made patients feel hesitant towards making decisions, and discussing the subject now.

### Is a treatment worth it?

The bottom layer in Fig. 2 is the patient’s consideration whether a treatment is worth it or not. This is the more practical perspective on care decisions that patients expressed. They mentioned care decisions is about the ‘balance between side effects and benefits of a treatment’, ‘to put the possibilities in context’, ‘to what extent you want to be treated and when you no longer want that’. It comprised thinking about whether something is worthwhile, and indicating boundaries if a treatment is not worthwhile. Several subthemes play a role in this balancing act, as will be described below.

#### Quality of life

Patients stated quality of life as an important factor in care decisions and the choice to limit treatments or not. Patients (or their relatives they use as an example) did not want ‘agony’, ‘a very heavy treatment process’ or to be treated when ‘it is hopeless’. They were afraid to become ‘a vegetable’ or were ‘reluctant to lose quality of life’. Life ‘should still be liveable’. They considered whether a treatment (and its side effects) is worse than the disease, ‘maybe live a little shorter but then you don’t have any misery because of the side effects’. Some stated to treat only if there is ‘hope’ for the future.

#### Being informed

Patients discussed that being informed of options and possibilities concerning care decisions is needed to be able to make decisions whether care is worthwhile or not. Currently they experienced a knowledge deficit, although the patient education did contribute to being informed. Especially the idea that care decisions include more than resuscitation and the background information given about other choices was mentioned as informative by many patients. Some stated being informed as a general benefit of the patient education, others mentioned specific positive consequences. For example, when one is aware of the options, one can form their own opinion and become enabled to make choices. One patient mentioned being less overwhelmed when a critical situation occurs if you have thought about care decisions in advance.

#### Patient in charge

Numerous patients indicated the importance of thinking about your own wishes. Some viewed thinking about care decisions as beneficial, because it empowers a patient to take control rather than depending on the input of the physician. Patients named ‘being in charge’, ‘taking responsibility’ and ‘being aware of your own wishes’ both as a benefit from the patient education and as a necessity to be able to discuss care decisions. To be able to be in charge, the patient must be informed, as can be seen by the quote of patient 41 in Table 1. Some patients stated they had to be ‘forced’ to take responsibility for their treatment and think about care decisions and said the patient education was beneficial in doing so.

#### Culture, family and loved ones

Patients mentioned two more important factors in care decision discussions and decisions: culture and family and loved ones. One patient, from a non-western culture, pointed out differences in directness and openness in communication and the role culture plays in care decisions. Some other patients mentioned the position of family and loved ones in care decision discussions, either as a reason not to make decisions beforehand (because a family member can fulfil this duty when necessary), or as a reason to indeed make decisions beforehand and discuss these with family (so they are aware of your opinion and not burdened with this task). Some stated it was something you should decide together with you family, as it concerns them as well.

### Physician

The final theme we identified is the physician. As is visualized in Fig. 2 by the wheels, the physician plays a key role in care decisions according to patients.

When we asked patients openly what they perceived as necessary or helpful in discussing care decisions, many patients mentioned factors related to the physician, and more specifically the physician’s communication. Words patient used to describe the desired communication style are for instance ‘honest’, ‘sensitive’, ‘clear’, ‘open’, ‘trustworthy’, ‘attention for the person’, ‘treating the patient as an equal’ or just ‘good communication’. This demonstrates the need for a sensitive and empathic communicative approach: physicians should take into account their communication style.

Another common opinion amongst patients is that the physician should initiate the care decision discussion. This was most often mentioned in relation to the question what would be necessary or helpful to discuss care decisions. Patients related this to the sensitivity of the topic, for instance, ‘because people are hesitant to think about it, it would be better if someone else starts talking about it’, or to the perceived current irrelevance, for instance ‘because people are not thinking about that yet’, ‘if the physician makes the assessment it is relevant for this person, then the physician should also take initiative’.

The physician is also assigned a task in informing the patient and resolving the knowledge deficit: ‘as a patient you don’t think of all the things that the physician can think of’. A minority of the patients fully trusted their physician’s expertise and preferred to leave the decision-making up to the physician. Most stated that the physician should inform them about treatment options, pros and cons, risks and chances of recovery: ‘if you choose not to do this, this is what it means, and also what it means if you choose to indeed do so’. The physicians role is not limited to informing. Patients also stated the physician should ‘make you really consider so really ask the questions’, in order to help the patient make up the balance. The physician can/ should enable the patient to take charge: ‘the patient should feel they have a choice (…) and they are free to make choices’, ‘the physician might know better, but the patient should know what he wishes’, ‘that the patient is made to think about it and that he dares to speak’. Finally, patients stated it is important that the physician is aware of their patient’s opinion, and is assigned the role as registrar of this opinion.

Because the physician is connected to almost all other (sub)themes, we visualised the physician as a wheel at the centre of Fig. 2, able to initiate and generate discussions, taking into account all themes perceived as relevant by the patients.

## Discussion

We aimed to gain deeper insight in patient’s perspective on the topic of care decisions. Most patients considered care decisions as belonging to the end-of-life, and therefore currently irrelevant. Consistent with other research, reading, talking or deciding about care decisions is perceived as unnecessary at this moment in time, because patients feel relatively healthy [15, 21–25].

There seems to be a vicious circle: literature about and research on care decisions is predominantly conducted in end-of-life settings. Furthermore, patients associate care decisions with the end-of-life. And in a previous study we showed that doctors frame the topic often as ‘relevant in the future’ as well [26]. Altogether, this results in postponement of the discussion of care decisions, and consequently research can only be conducted in the end-of-life phase. As is recognised by the Dutch Association of Internal Medicine by incorporating it in the Choosing Wisely campaign, this cycle should be broken, otherwise care decision conversations keep being assessed as being too soon, until it is too late [1].

We attempted to address this perceived irrelevance in our current patient education by emphasizing the current importance of care decision conversations. However, our study shows this attempt was insufficient. Several behavioural models have described differences in information processing and likelihood of persuasion depending on motivation [27, 28]. Probably, the relevance and thereby motivation to process information about care decisions should be even further emphasized. In order to break the vicious cycle, we might need to do more than patient education alone.

Another important connection patients made with the topic of care decisions was the need to make definite, binding decisions. This created a barrier, because patients expressed that the balance whether a treatment is ‘worth it’ depends on the situation. This barrier corresponds with previous research [29, 30]. Care decision discussions should not focus on fixed decisions, but on goals of care and the regular discussion of treatment options and preferences, as it better fits patient’s changing needs [30]. Our research shows patients should be aware of that as well.

Very recently, Harris et al. conducted a qualitative interview study on goals of care discussions in acute hospital admissions in Australia [15]. Although their study population differed from ours (experiences of acute hospitalized patients with goals of care discussions versus the perspective of an outpatient population), we found many similarities. Both patient populations perceived care discussion irrelevant at this moment in time. Also, they described the connection to dying and death, a focus on resuscitation, a knowledge deficit, and the need for involvement of family.

There are several strengths and limitations to our study. The qualitative approach and semi-structured interviews provided us the ability and flexibility to get in-depth information about aspects of patients’ perspective on the topic of care decisions. In line with the growing awareness that care decision discussions should take place ‘earlier’, we investigated the perspective of the, relatively healthy, internal medicine outpatient clinic population. In this study, the median age was 57,5 years (interquartile range 53–67,5) and the patients had a median Charlson Comorbidity Index of 2,5 (interquartile range 1–4), which means they were relatively healthy and not in the end of life. This adds to existing research which mostly revolves around patients with severe chronic diseases, elderly patients or patients with a terminal illness [8, 15].

We are aware that in an interview-study the ways in which questions are asked have an effect on the patient responses and can thus have an effect on the themes that are identified. To minimize this risk, we mostly asked open questions. For instance, all physician-related factors patients mentioned, were an answer to “what is necessary/ helpful in care decision discussions”. We did not ask “what should the physician do” or “what is the role of the physician” (which inevitably would have resulted in a theme physician).

Another potential limitation arises from the notable amount of eligible patients that did not answer the phone or did not want to participate in the interviews, as this might originate from a certain perspective or emotional response to the topic of care decisions. However, numerous participants expressed hesitance regarding the topic, which pleads against this group being underrepresented. Furthermore, patients with insufficient command of the Dutch language could not participate in this study. One patient (from a non-western culture), pointed out some cultural differences, but we have too little data to draw conclusions on cultural differences. Lastly, the amount of time between reading the patient education and the interview varied between patients, and some were unable to remember the content very well. However, in a normal clinical setting, patients would not read a patient education on a daily basis either, and their perspective on care decisions was still insightful.

## Conclusion

This study showed that patients’ perception of the topic of care decisions is overshadowed by two (wrongful) convictions: the perception that it belongs to the end-of-life and therefore is not relevant for them at this moment in time, and the belief that care decision discussions leads to fixed decisions. This resulted in assessing our patient education as informative, but not helpful at this moment in time and no desire to discuss care decisions yet.

### Future perspective

Our research shows some opportunities to improve care decision discussions. The top layer in Fig. 2 shows two deep convictions patients have, that prevents them from going to the actual, more practically orientated, balancing whether a treatment is ‘worth it’. These two associations, with the end-of-life and need for binding decisions, should be addressed first. These convictions seem persistent and call for a change of the care decision narrative. We propose care decisions should be a normal, regular, recurring part of the medical consultation. This “new” narrative, of care decision conversations as a continuous, dynamic process, relevant at any given time and circumstance, should be disseminated. Framing it as “a plan” could possibly be helpful in seeing it as currently relevant and flexible, rather than fixed. To accomplish this, both patient, physicians, and perhaps even society should be informed and engaged. One might think of patient education, a short informative movie in the waiting room, or even a national campaign. Patients assign the physician a key role in this process, so the physician should pick up the gauntlet and take this role. Physicians should be educated in this role, and specifically in the expectation of patients that the physician initiates this conversation, informs them, and does so with sensitive communication skills. As a regular part of the medical consultation.

### Electronic supplementary material

Below is the link to the electronic supplementary material.

**Supplementary Material 1**: Supplementary appendix 1: COREQ checklist

**Supplementary Material 2**: Supplementary appendix 2: Interview guides

## Data Availability

Raw data (videos and transcripts) are not publicly available to preserve participants’ privacy. On reasonable request, data can be requested from the first author, S. Briedé.

## Declarations

### Author agreement

We declare that all authors have seen and approved the final version of the manuscript being submitted. The article is the authors’ original work, hasn’t received prior publication and isn’t under consideration for publication elsewhere.
