# Supplementary material for: A patient’s perspective on care decisions: a qualitative interview study
Source: BMC Health Serv Res. 2023 Dec 1;23:1335. doi: 10.1186/s12913-023-10342-9 (PMC10693144; doi:10.1186/s12913-023-10342-9)
Supplement: Supplementary file 1 — Supplementary Material 1: Supplementary appendix 1: COREQ checklist [file 12913_2023_10342_MOESM1_ESM.docx]

| **No** | **Item** | **Response** |
| --- | --- | --- |
| **Domain 1: Research team and reflexivity** | | |
| *Personal Characteristics* | | |
| 1. | Interviewer/ facilitator | The interviews were conducted by AB and NB after training and under supervision of SB. |
| 2. | Credentials | SB: medical doctor; NB: medical student; TvC: PhD, assistant professor language and communication; KK: medical doctor, professor of Acute Internal Medicine; AB: medical student. |
| 3. | Occupation | SB is a resident in internal medicine at the University Medical Centre Utrecht and PhD student at Utrecht University. TvC is assistant professor language and communication at the department of Languages, Literature and Communication at Utrecht University. KK is professor of Acute Internal Medicine at Utrecht University. NB and AB were medical students at Utrecht University during the conduct of the study, currently NB is a medical doctor. |
| 4. | Gender | All researchers were female. |
| 5. | Experience and training | SB is a PhD student and therefore received formal education in research and medical ethics. KK is professor of acute internal medicine with research experience mainly in quantitative research. TC is assistant professor language and communication. Her research is mostly concerned with talk in interaction. She focuses mainly on talk in the health domain. SB and KK are both BROK certified (Basic course on Regulations and Organisation for clinical investigators). Before this project, SB was trained in qualitative research and especially interview studies by dr. A Oerlemans, assistant professor IQ Healthcare at Radboud university medical centre. NB and AB followed a Good Clinical Practice course and works under supervision of SB. |
| *Relationship with participants* | | |
| 6. | Relationship established | The interviewers did not have a prior relationship with the participants. The interviewers were not involved in the recruitment of patients in the previous study or the distribution of the patient education. |
| 7. | Participant knowledge of the interviewer | Participants were aware the interviewers and other researchers were conducting a research project with the aim to understand more about the participants perspective on treatment wishes and limitations and the assessment of the patient education. |
| 8. | Interviewer characteristics | The participants were aware the interviewers were medical students during a research internship and the aim of the research team was to publish this research data. |
| **Domain 2: study design** | | |
| *Theoretical framework* | | |
| 9. | Methodological orientation and Theory | Collected data was analysed using reflexive thematic analysis with an inductive approach. Throughout the process, we operated within a qualitative paradigm, corresponding to the “Big Q thematic analysis” described by Terry et al. [17] |
| *Participant selection* | | |
| 10. | Sampling | Participants were selected using convenience sampling. Participants that had received the patient education in a clinical setting (i.e. the intervention group of the previous study) were approached for participation. |
| 11. | Method of approach | Participants were approached by telephone. |
| 12. | Sample size | 44 participants (34 in the first round, 10 in the second round) |
| 13. | Non-participation | Of the 62 patients that were approached for this study, 13 did not want to participate, 3 could not be reached, and 2 did not answer the telephone at the scheduled interview time. (See figure 1). |
| *Setting* | | |
| 14. | Setting of data collection | Data was collected by telephone. |
| 15. | Presence of non-participants | No non-participants were present. |
| 16. | Description of sample | Of the 44 patients, 25 were male (56,8%) and 19 female (43,2%). The median age was 57,5 years (interquartile range 53-67,5) and they had a median Charlson Comorbidity Index of 2,5 (interquartile range 1-4) |
| 17. | Interview guide | We provided the interview guides (appendix 2) |
| *Data collection* | | |
| 18. | Repeat interviews | No repeat interviews were carried out. |
| 19. | Audio/visual recording | Interviews were audio-recorded, downloaded onto a secure folder at the UMC Utrecht network, and subsequently deleted from the recording device. The interviews were transcribed by NB and AB and checked by SB for accuracy. |
| 20. | Field notes | No field noted were collected. |
| 21. | Duration | Interview duration was on average 15-20 minutes. |
| 22. | Data saturation | Not applicable. |
| 23. | Transcripts returned | Transcripts were not returned to participants. |
| **Domain 3: analysis and findings** | | |
| *Data analysis* | | |
| 24. | Number of data coders | Two of the researchers (SB and NB) coded the data. |
| 25. | Description of the coding tree | No coding tree was used as subthemes and themes were inductively derived from the data. |
| 26. | Derivation of themes | Themes were derived from the data. |
| 27. | Software | NVivo 12 software |
| 28. | Participant checking | Participants did not provide feedback on the findings. |
| *Reporting* | | |
| 29. | Quotations presented | Table 1 shows illustrative quotations for each theme with unique study numbers for participant identification. Besides, in the text short quotes are shown for additional illustration. |
| 30. | Data and findings consistent | According to the research team data presented and findings are consistent. |
| 31. | Clarity of major themes | We identified 4 themes and 10 subthemes. Figure 1 shows the relationship between the themes. |
| 32. | Clarity of minor themes | Not applicable |
