# Supplementary material for: A patient’s perspective on care decisions: a qualitative interview study
Source: BMC Health Serv Res. 2023 Dec 1;23:1335. doi: 10.1186/s12913-023-10342-9 (PMC10693144; doi:10.1186/s12913-023-10342-9)
Supplement: Supplementary file 2 — Supplementary Material 2: Supplementary appendix 2: Interview guides [file 12913_2023_10342_MOESM2_ESM.docx]

**Appendix 2: Interview guides**

*First round of interviews*

Topics:

1. Time spent on the patient education
2. Design
3. Helpfulness, why of why not?
4. Positive aspects
5. Missing information
6. Unnecessary information
7. Opinion about distributing the patient education
8. Necessities to discuss treatment wishes and limitations
9. Questions, things to add or comments?

*Second round of interviews*

Topics:

1. What comes to mind when hearing the term treatment wishes and limitations?
   - Why? What makes it provoking these feelings? What could change that?
2. Experiences with the patient education
3. Have you ever discussed treatment wishes and limitations?
   - With whom? How did you experience that?
4. Feelings about discussing treatment wishes and limitations
5. Relevance of treatment wishes and limitations, why or why not? If not, when would it be relevant?
6. What could aid discussing treatment wishes and limitations?
7. Questions, things to add or comments?
